# The Evolving Role and Impact of Integrating Pharmacists into Primary Care Teams: Experience from Ontario, Canada

**DOI:** 10.3390/pharmacy8040234

**Published:** 2020-12-07

**Authors:** Manmeet Khaira, Annalise Mathers, Nichelle Benny Gerard, Lisa Dolovich

**Affiliations:** 1 Leslie Dan Faculty of Pharmacy, University of Toronto, 144 College St, Toronto, ON M5S 3M2, Canada; manmeet.khaira@hotmail.com (M.K.); annalise.mathers@utoronto.ca (A.M.); nichelle.bennygerard@mail.utoronto.ca (N.B.G.); 2 School of Pharmacy, University of Waterloo, 10 Victoria St S, Kitchener, ON N2G 1C5, Canada; 3 Department of Family Medicine, McMaster University, 1280 Main St W, Hamilton, ON L8S 4L8, Canada

**Keywords:** primary care team pharmacist, pharmacist-physician collaboration, comprehensive medication management, chronic care management, team-based primary care, interprofessional collaboration

## Abstract

The movement to integrate pharmacists into primary care team-based settings is growing in countries such as Canada, the United States, the United Kingdom, and Australia. In the province of Ontario in Canada, almost 200 pharmacists have positions within interdisciplinary primary care team settings, including Family Health Teams and Community Health Centers. This article provides a narrative review of the evolving roles of pharmacists working in primary care teams, with a focus on evidence from Ontario, as well as drawing from other jurisdictions around the world. Pharmacists within primary care teams are uniquely positioned to facilitate the expansion of the pharmacist’s scope of practice, through a collaborative care model that leverages, integrates, and transforms the medication expertise of pharmacists into a reliable asset and resource for physicians, as well as improves the health outcomes for patients and optimizes healthcare utilization.

## 1. Introduction

Although pharmacists are the most accessible and visited healthcare professionals in the world, the contributions that pharmacists make to interdisciplinary healthcare settings often remain overlooked [1,2,3]. It is known that poor communication and connectivity between healthcare professionals can fragment patient care, is a significant contributor to the development of drug-related problems (DRPs), and results in poorer health outcomes and experiences [4,5,6]. Moreover, since most prescribing of medications occurs in primary care, defined as a “whole-of-society approach to health and well-being” that addresses the broader determinants of health [7], pharmacists have an integral role in providing education and information about the appropriate and safe use of medications to patients, as well as to other healthcare professionals [8]. Healthcare professionals who work within a primary care team (PCT) have significantly improved communication and coordination, are optimally placed to detect and resolve DRPs, and can improve the availability and efficiency of healthcare [9,10].

In the past two decades, the movement to include pharmacists as essential members of PCTs has gained traction in a number of countries, including Canada [9,11,12], the United States [13,14,15], the United Kingdom [16,17], Australia [18], Malaysia [19,20,21], and Brazil [22]. Pharmacists integrated into interdisciplinary PCTs globally demonstrated their significant role in many direct patient care activities, including medication management, identifying adverse or incorrect medication usage, counselling on medications, and effectively optimizing a patient’s understanding of their own medication regimens to enhance overall quality of life [9,23].

Canada has 10 provinces and 3 territories in which healthcare is primarily the responsibility of the province. In the province of Ontario, this is achieved with connection and coordination of the provincial healthcare system carried out by Ontario Health, an agency created by the Government of Ontario. Ontario recently transitioned to a regional health administrative structure with the introduction of Ontario Health Teams, which will eventually provide healthcare coverage for different areas of the province. Each Ontario Health Team is expected to provide integrated care across healthcare sectors, within a local community, including primary care. Primary care is provided through a mix of fee-for-service and organized-team-based care models. The most cohesive and comprehensive team-based care models are Family Health Teams (FHTs) and Community Health Centers. These provide care for approximately 20%, or 3 million people, in the province and are the main team-based setting, which include integrated primary care team pharmacists [24].

There are currently over 15,000 pharmacists working in Ontario (comprising approximately one-third of all pharmacists in Canada) [25] in pharmacy settings, including community, hospital, and interdisciplinary PCTs, such as FHTs and Community Health Centres [26]. Pharmacists are included as healthcare team members since the inception of the FHT model in Ontario in 2005. In FHTs, pharmacists provide on-site and in-office care to patients through specialized clinic services and comprehensive medication therapy management, and patient documentation by a pharmacist is included in the site electronic medical record [27]. Additionally within Canada, the province of British Columbia recently initiated a pilot program in 2018 to eventually integrate 50 pharmacists into PCTs, with the goal of revolutionizing pharmacy practice and patient care in that province [28]. Despite recent steps to embed pharmacists across Canada into PCTs, Canada lags behind other countries like the United Kingdom, where the National Health Service implemented a $100 million (in United States Dollars), five-year program to incorporate 1500 pharmacists into PCTs [17].

Given the continued expansion of pharmacy scope of practice in Ontario and across Canada, this article aims to provide a narrative review describing the evolution and evidence of the role of PCT pharmacists in Ontario, and then considers this evidence for the current impact of PCT pharmacists, in other jurisdictions around the world.

## 2. The Pharmacist’s Role in Primary Care

The skills of pharmacists in primary care include the provision of direct patient care through management of medications, examination and screening, chronic disease management, drug information and education, collaboration and liaison, quality assurance, and research [29,30,31].

A study surveying pharmacists working in Ontario FHT sites about their involvement in various primary care activities found that the majority of time was spent on managing medications, conducting medication reviews, and communicating and educating other healthcare professionals (Table 1). As Ontario continues to see an expanded scope of practice, further research is required to determine how the activities of pharmacists in PCTs will also evolve.

In contrast to other pharmacy settings, pharmacists working in PCTs have additional roles that emerge when working with other healthcare professionals [33]. For example, pharmacists working in Ontario FHTs reported that their role was more strongly anchored in supporting healthcare professionals to manage medication use, locally implement national health priorities, arrange access to funding and health services, as well as design treatment pathways for patients [34]. The wider scope of a pharmacist’s clinical duties in PCTs, which can include point of care anticoagulation monitoring in specialty PCT clinics, and direct collaboration with other healthcare professionals was shown to result in a longer acclimatization process for pharmacists in FHTs across Ontario, than for pharmacists in other practice settings [35]. However, research in Canada and the United Kingdom suggests that pharmacists working in primary care are well-positioned to build relationships with pharmacists working in community and hospital settings and ultimately collaborate to provide patient care that is coordinated across pharmacy settings. This might include monitoring of patients started on new medications, patients transitioning between healthcare environments, and improved accuracy and continuity of medication review assessments, and other healthcare information [36,37]. These intra-professional relationships and roles are critical to facilitate improved patient care. In the United Kingdom, a pilot project integrating nearly 1500 pharmacists into PCTs highlighted the additional roles of pharmacists responding to hospital discharges and prescribing, which, unlike in many other countries, pharmacists have the authority to do in the United Kingdom [38].

## 3. The Demonstrated Value of Pharmacists Integrated into PCTs

There is increasing evidence and support for the broader integration of pharmacists into PCTs. In Ontario, pharmacist integration into PCTs was shown to improve an array of health outcome quality measures, such as appropriate medication use, hypertension control, medication consultations, improved prescribing, reduced healthcare utilization and medication costs, and improvement in the management of chronic conditions [37]. The reductions in incorrect or unsafe use of medications quantify the direct benefits of the pharmacist’s role into interdisciplinary PCTs and are further supported by research in Ontario, where pharmacists reported improved confidence in their role and the ability to support other healthcare professionals [10].

Identification of DRPs is one of the most significant contributions a PCT pharmacist can make. Research conducted across Ontario FHTs showed that medication reviews conducted by pharmacists are able to reduce medication discrepancies and rectify DRPs [6]. In this study, among a sample of 237 patients, 16 pharmacists found that patients were on an average of 9.2 prescription medications, and identified an average of 2.1 medication discrepancies and 3.6 DRPs per patient [6]. Pharmacists identified more than one medication discrepancy per patient and that almost every patient had a drug therapy problem. Similarly, in another Ontario study, when 7 pharmacists were integrated into seven PCTs and conducted medication reviews for 969 patients, PCT pharmacists were able to identify at least one DRP in 93.8% of patients, and also found an average of 4.4 DRPs per patient [37].

Another study of 1634 patients in an Ontario FHT identified DRPs in 88% of patients, with the most common DRPs categorized as additional drug therapy needed (33.7%), inappropriate dosage (16.1%), and adverse drug reactions (13.7%) [39]. When DRPs were identified by the PCT pharmacist, a positive clinical outcome was realized for approximately 80% of patients, upon 2-year follow-up [32]. These findings are comparable to findings across FHTs in Ontario that found the most common DRPs were additional drug therapy needed (22.6%), inappropriate dosage (14.1%), and receiving a drug with no indication (13.1%) [6]. In the province of Quebec, one study reported that pharmacists detected 300 DRPs (an average of 7.2 per patient) and that the most common DRP was ‘drug use without indication’ (27%); physicians accepted nearly 90% of the recommendations made by PCT pharmacists [9]. This is mirrored in Australia where PCT pharmacists have a higher rate of recommendations made and implemented, as a result of medication reviews, than those conducted by non-PCT pharmacists [40,41]. In another Australian study, the number of DRPs per patient fell to zero, after a 6-month follow-up with a PCT pharmacist, while patient adherence to medications simultaneously improved by approximately 20% [5]. These findings are further supported by a randomized control trial in Malaysia for patients with diabetes, hypertension, and hyperlipidemia, in which pharmacists were able to identify a medication issue in over 50% of patients [21]. Pharmacists were able to convey these issues to physicians, who implemented 87% of the pharmacist’s recommendations [21]. Collectively, these findings demonstrate that pharmacists in PCTs are able to identify and address medication discrepancies and DRPs, to improve medication management, the provision of appropriate prescribing and simplifying patient’s medication regimens.

The impact of pharmacist-led interventions in PCTs for elderly patients also improved medication adherence and reduced emergency room visits and hospitalizations due to DRPs [32], as well as improved prescribing appropriateness [42] in Ontario and globally. These improvements are particularly significant among polypharmacy patients [43]. Pharmacists in Ontario-based PCTs also showed improvements for chronic condition management among patients on medications for diabetes [44] and anticoagulation [35], which is also complemented by additional evidence from the province of Alberta, Canada, for improvements in blood pressure and cholesterol [45]. These studies demonstrate the pivotal and proactive role pharmacists play in optimizing patient care, when integrated into PCT settings around the world.

## 4. Collaboration with Physicians

Physicians attributed many benefits to having a pharmacist integrated into their practice, including having a colleague who is able to provide reliable drug information, optimize medication prescribing, improve clinical documentation, services, and recommendations, and enhance patient care [46]. In research conducted among physicians working in PCT sites across Ontario, physicians are supportive of receiving, and choosing to implement recommendations made by pharmacists working at the same PCT site [47]. In turn, PCT pharmacists reported receiving more consultations and referrals once the physician was offered initial feedback and suggestions regarding treatment plans for their patients [46,47,48]. At Women’s College Hospital in Toronto, collaboration between a clinical pharmacist, a pharmacy resident, and physicians, allowed the interdisciplinary team to successfully meet their deprescribing goal, aligned with the World Health Organization’s call for action to decrease avoidable medication-related harm [49]. This collaboration between physicians and pharmacists also led to improved relationships and communication between healthcare professionals that facilitate patient care planning, documentation, and implementation [33].

In the state of California in the United States, 69 physicians participated in a cross-sectional survey on their experience working with clinical pharmacists in PCTs; 90% of physicians noted improved medication management of their patients and 93% of respondents recognized the pharmacist’s recommendations as clinically meaningful [15]. Similarly, in a survey of fifty-six physicians in the state of North Carolina, 87.5% felt that identifying enhanced clinical outcomes was the top benefit of embedding a clinical pharmacist into their practice [50]. In addition to optimizing outcomes for patients, a systematic review found that pharmacists in PCTs reduced physician workload substantially [51]. Primary care physicians further described that the inclusion of a clinical pharmacist can improve the perceived quality of their patients’ healthcare, the quality of the medication decisions made, and the management of medications [29]. Patients who met with both a physician and pharmacist when transitioning between points of care, for example, from hospital to home, reported a reduced hospital readmission rate as well as benefitted from discontinuing an unnecessary medication, receiving new medications, and having non-adherence addressed [29].

In jurisdictions such as Malaysia where pharmacist integration is emerging, it is important to leverage evidence from countries where integration of pharmacists was successful, in order to strengthen physician awareness of and support for the significant roles and contributions of pharmacists in PCTs [19,20].

## 5. Perspectives from Patients

The benefit of pharmacists in primary healthcare was also studied among patients. At the Centre for Family Medicine Family Health Team in Ontario, patients and caregivers communicated the benefits of having a comprehensive medication review in an interdisciplinary setting. This includes a decrease in inappropriate medication use, optimization of hypertension and diabetes medication regimens, as well as minimization of antipsychotic use for the behavioral and psychiatric symptoms of dementia [33]. In the United States, pharmacist-managed clinics act as an opportunity for patients to receive detailed medication information, focused on their specific needs and desires [52]. Patients expressed a sense of companionship with the pharmacist, which improved the patient’s desire to reach their healthcare goals [52]. In Malaysia, qualitative research on patient perspectives on pharmacist integration demonstrates that patients believe pharmacists play a substantial role in informing patients of the safe and appropriate use of medications [19]. Additionally, when PCT pharmacists provide medication education and information, many patients feel that the medication-related education, disease-related education, and delivery of education they receive is excellent [53]. Research around the world supports that patients prefer to have their care coordinated between a physician and pharmacist and recognize that this collaboration is integral to optimizing their care [5].

## 6. Evolving Pharmacy Education and Practice

The role, impact, and value that pharmacists contribute to primary care is significant. In order to support the continued integration of pharmacists into PCTs, improvements in educational and training opportunities for young pharmacists is required globally [10,22]. The “ADapting pharmacists’ skills and Approaches to maximize Patient’s drug Therapy effectiveness” (ADAPT) program was developed based on experience with training and mentoring pharmacists, to integrate into PCTs in Ontario and that hosted by the Canadian Pharmacists Association [54,55,56,57]. The ADAPT program aimed to provide a standard approach to medication assessment, team collaboration, patient assessment, evidence-based decision making, and documentation, facilitated through an e-learning program [57,58,59]. This program provided evidence supporting continuing education via online learning, with pharmacists reporting high satisfaction and confidence in skills that they could directly apply in their professional careers [57,58]. The ADAPT educational program was adapted for use by clinical pharmacists and professors at the Virginia Commonwealth University School of Pharmacy in the state of Virginia, United States. Its use resulted in improvements in providing care, interviewing, documenting, and collaboration for pharmacists working within primary care in Canada and the United States [60].

Research from the existing Primary Care Pharmacy Specialty Network (PC-PSN) in Canada also demonstrated that listservs can act as key channels for PCT pharmacists to connect to share information, identify solutions for complex patients and care, and provide mentorship opportunities [61]. Further research demonstrates that pharmacists identified key competencies for working in primary care, which include a focus on communication, collaboration, and professionalism, and consider how these relate to pharmacists and other healthcare professionals in understanding the evolving roles of PCT pharmacists in order to establish performance indicators to support professional education [62]. As found in Brazil, training opportunities within interprofessional teams also improves the understanding that healthcare professionals have about the roles and competencies of pharmacists on their PCT, and in the long-term can help to demonstrate the impact and importance of their work [22]. To complement this work, opportunities for pharmacists to gain direct experience through training and educational placements in PCT environments, and to continue to build a community that fosters sharing of interprofessional clinical knowledge and skills, will better equip pharmacists for future practice in PCT environments.

To strengthen the successful integration of pharmacists into PCT practice settings, is it critical to ensure there are opportunities and support available for the increased visibility of pharmacists as PCT ambassadors. For example, in Ontario, at times a pharmacist represents allied healthcare professionals on the Association of Family Health Teams Ontario (AFHTO) Board of Directors, which improves the visibility and credibility for the role of pharmacists in PCTs [26]. Furthermore, the Pharmacy Specialty Network (PSN) developed by the Canadian Society of Hospital Pharmacists and the Canadian Pharmacists Association, enables pharmacists to share practice-based resources; develop, support, and maintain networking opportunities for pharmacists; advocate for the role of pharmacists in PCTs; and provide education and training to its members, including mentorship opportunities for pharmacists new to PCT settings [63]. The development of Pharmacist Program Toolkits by the IMPACT project (Integrating Family Medicine and Pharmacy to Advance Primary Care Therapeutics; www.impactteam.info) provides guidance and strategies for PCTs to successfully integrate pharmacists alongside other healthcare professionals. To provide additional resources for PCT pharmacists, the Ontario Pharmacists Association developed a toolkit [64] that pharmacists can leverage to practice to their full scope.

## 7. Improved Outcomes for Patients and Optimization of Health Systems

A systematic review found that emergency room visits decreased and savings in medication and health system costs were realized when pharmacists are integrated into PCTs, even with increased primary care usage [51]. Improvements in health outcomes at the individual and population level was also demonstrated when pharmacists work as a member of an inter-professional team, as well as decreased fragmentation within the healthcare system [51]. Although there are no published economics analyses based on the Ontario data, one study estimates that PCT pharmacists can offset costs to the healthcare system by $1079 per patient and can generate revenues for the PCT that are 38% in excess of the cost of the pharmacist’s time [29]. To realize these opportunities more fully in primary care in Ontario and around the world, further research is required to evaluate health system impacts and outcomes, over the long-term, upon embedding pharmacists into PCTs.

## 8. Conclusions

Pharmacists in Ontario are now formally funded by the public healthcare system to be members of the interdisciplinary healthcare team. There is a growing evidence base that describes the role and impact of Ontario-based PCT pharmacists, which is consistent with evidence emerging worldwide. Pharmacists within PCTs are uniquely positioned to facilitate the expansion of the pharmacist’s scope of practice through a collaborative care model that leverages, integrates, and transforms the medication expertise of pharmacists into a reliable asset and resource for physicians. Further research in Ontario is needed to quantify the effectiveness of PCT pharmacists on health outcomes, and the resulting impact on healthcare service use and costs.

## Figures and Tables

**Table 1:** Selected major activities performed by pharmacists in Family Health Teams (FHTs) in Ontario ^i^.

Activity Category	Specific Activity Carried Out	Percent of Respondents N: % (N = 70)
Direct Patient Care	Managing medication-related issues	96
	General medication reviews	70
	Medication reconciliation	63
Education and drug information	Unstructured education to other healthcare practitioners	73
	Mentoring students	27
	Structured education to patients in a group setting	16
Systematic improvement programs	Creating new programs for patients	19
	Improvement in drug prescribing/use	17
Other activities	Research/quality improvement	9

^i^ Adapted from [32].
